# High Potency of *Melaleuca alternifolia* Essential Oil against Multi-Drug Resistant Gram-Negative Bacteria and Methicillin-Resistant *Staphylococcus aureus*

**DOI:** 10.3390/molecules23102584

**Published:** 2018-10-09

**Authors:** Alessandra Oliva, Silvia Costantini, Massimiliano De Angelis, Stefania Garzoli, Mijat Božović, Maria Teresa Mascellino, Vincenzo Vullo, Rino Ragno

**Affiliations:** 1 Department of Public Health and Infectious Diseases, Sapienza University of Rome, Viale del Policlinico 155, 00161 Rome, Italy; silviacostantini89@gmail.com (S.C.); massimiliano.deangelis@uniroma1.it (M.D.A.); mariateresa.mascellino@uniroma1.it (M.T.M.); vincenzo.vullo@uniroma1.it (V.V.); 2 Department of Drug Chemistry and Technology, Sapienza University, P.le Aldo Moro 5, 00185 Rome, Italy; stefania.garzoli@uniroma1.it; 3 Faculty of Natural Sciences and Mathematics, University of Montenegro, Džordža Vašingtona bb, 81000 Podgorica, Montenegro; mijatboz@gmail.com; 4 Rome Center for Molecular Design, Department of Drug Chemistry and Technology, Sapienza University, P.le Aldo Moro 5, 00185 Rome, Italy; 5 Alchemical Dynamics s.r.l., 00125 Rome, Italy

**Keywords:** multi-drug resistant bacteria, essential oils, *Melaleuca alternifolia*, methicillin-resistant *Staphylococcus aureus*, carbapenem-resistant microorganisms

## Abstract

Purpose: Herein, an extended investigation of Tea tree oil (TTO) against a number of multi-drug resistant (MDR) microorganisms in liquid and vapor phases is reported. Methods: The activity of TTO was tested against methicillin-sensitive *Staphylococcus aureus* (MSSA), *Escherichia coli*, and clinical strains of methicillin-resistant *S. aureus* (MRSA), extended-spectrum beta lactamases producer carbapenem-sensitive *Klebsiella pneumoniae* (ESBL-CS-Kp), carbapenem-resistant *K. pneumoniae* (CR-Kp), *Acinetobacter baumannii* (CR-Ab), and *Pseudomonas aeruginosa* (CR-Pa). Minimal inhibitory/bactericidal concentrations (MIC/MBCs) and synergistic activity between TTO and different antimicrobials were determined. In the vapor assay (VP), TTO-impregnated discs were placed on the lid of a petri dish and incubated for 24 h at 37 °C. Results: TTO showed a potent bactericidal activity against all the tested microorganisms. TTO in combination with each reference antimicrobial showed a high level of synergism at sub-inhibitory concentrations, particularly with oxacillin (OXA) against MRSA. The VP assay showed high activity of TTO against CR-Ab. Conclusion: Evaluation of in-vitro activity clearly indicated TTO as a potential effective antimicrobial treatment either alone or in association with known drugs against MDR. Therefore, TTO could represent the basis for a possible role in non-conventional regimens against *S. aureus* and Gram-negative MDR. TTO in VP might represent a promising option for local therapy of pneumonia caused by CR-Ab.

## 1. Introduction

The emergence of multidrug-resistant (MDR) microorganisms represents a global challenge worldwide, since therapeutic options are limited, resulting in thousands of deaths [1]. Furthermore, there is a paucity of novel and effective antimicrobial agents in the pipeline, especially against carbapenem-resistant (CR) *Acinetobacter baumannii* and CR *Enterobacteriaceae* carrying enzymes other than class A carbapenemases [2].

Essential oils (EOs) are volatile, natural, fragrant liquids that can be extracted from different parts of the plants (especially leaves and flowers) presenting anti-inflammatory, antiviral, and antibacterial properties [3]. Given their antimicrobial activity broad-spectrum, together with the possibility of restoring antibiotic susceptibility [4,5], several efforts have been made to consider the use of EOs for the treatment of a wide range of infections, including those caused by MDR microorganisms [6]. EOs’ activity is commonly ascribed to the perturbation of cell membrane structural integrity, leading bacterial cell to death [7] and their potency varies with the type of microorganisms, Gram-positive bacteria being more susceptible than Gram-negatives [8,9,10,11].

Tea tree oil (TTO) is produced by steam distillation of leaves and terminal branches of *Melaleuca alternifolia* and is currently used in traditional medicine as a topical antiseptic and anti-inflammatory agent and widely formulated into many cosmetic and personal care products [12]. TTO is mainly known for its antibacterial properties [13], exerted by the inhibition of bacterial respiration and the disruption of the permeability barrier of microbial membrane structures, as well as by the induction of a leakage of potassium ions, both in Gram-positive and Gram-negative bacteria [14]; in addition, it has been widely investigated in synergy with conventional antimicrobials, such as vancomycin [15] and aminoglycosides for *S. aureus* and *E. coli* [16] and, recently, combined with different nanoparticles [17]. However, little is known with regard to the activity of TTO against MDR and pan-drug resistant (PDR) Gram-negative strains both in liquid and VP as well as the potential interaction (i.e., restoring antibiotic sensitivity) between beta-lactams and TTO towards MRSA.

Based on the above considerations and continuing the investigations on EOs as effective antimicrobial agents [10,18,19,20,21,22,23], the main aim of this report was to evaluate the in-vitro activity of a chemically characterized commercial TTO, alone and in combination with different antimicrobials, against methicillin-susceptible *Staphylococus aureus* (MSSA), methicillin-resistant *Staphylococus aureus* (MRSA), *Escherichia coli* and MDR Gram-negative bacteria including extended-spectrum beta lactamases (ESBLs) producer carbapenem-sensitive *Klebsiella pneumoniae* (ESBL-CS-Kp), ESBL and carbapenem-resistant *K. pneumoniae* (CR-Kp), carbapenem-resistant *Acinetobacter baumannii* (CR-Ab) and carbapenem-resistant *Pseudomonas aeruginosa* (CR-Pa). In addition, the antibacterial effectiveness of TTO in the VP against the abovementioned microorganisms was investigated.

## 2. Results

### 2.1. TTO Chemical Characterization

Chemical TTO characterization was performed throughout gas chromatographic/mass spectrometric (GC/MS) analysis both in the liquid and vapor phase (VP).

Among the several chemical components contained in the TTO sample (Table 1), terpinen 4-ol, eucalyptol, α-pinene, and γ-terpinene were found to be the most abundant by the classical GC/MS analysis performed on the liquid EO (35.4%, 15.2%, 12.4%, 9.8%, respectively). A different scenario was instead observed by performing the EO VP analysis through headspace technique. A direct comparison of the two analyses indicated a marked increase in the percentage of α-pinene (22.5%) and almost 20% reduction in terpinen 4-ol (28.7%) in the VP.

### 2.2. Antimicrobial Susceptibility

TTO minimal inhibitory/bactericidal concentrations (MIC/MBCs, respectively) were evaluated against a list of standard and clinical isolate strains of important microorganisms such as MSSA, *E. coli*, MRSA, ESBL-CS-Kp, CR-Ab, and CR-Pa. AMK, CFZ, MEM, OXA, COL, RIF and VAN were used as references drugs (Table 2). As expected, CR strains exhibited high MEM MIC/MBC values whereas both MSSA and MRSA showed sensitivity to VAN and RIF.

TTO showed a potent bactericidal activity (expressed in *v*/*v* percentage) against all the tested Gram-negative microorganisms, with MIC/MBCs 0.25%/0.25% for CR-Ab, CR-Kp and *E. coli*, 1%/1% for CR-Pa, 0.5%/0.5% for ESBL-CS-Kp. With regard to MSSA and MRSA, MIC/MBCs were 1%/2% and 0.5%/2%, respectively. For all the tested microorganisms, the MBC resulted in absence of bacterial growth after 24 h of incubation.

### 2.3. Synergistic Activity

TTO in combination with each reference antimicrobial showed a high level of synergism at sub-inhibitory concentrations, especially with CFZ/OXA/AMK against both MSSA and MRSA and with MEM/AMK/COL against all Gram-negative microorganisms (Table 3).

Notably, TTO at sub-inhibitory concentrations lowered OXA and CFZ MICs for MRSA from 64 to 2 μg/mL and from 32 to 1 μg/mL, respectively.

### 2.4. Disk Diffusion (DD) and VP Assay

**Figure 1:**
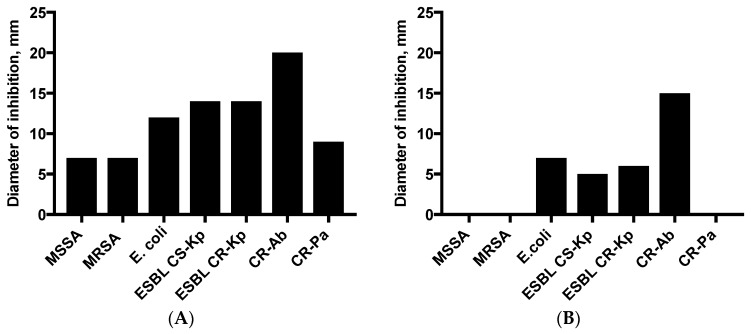
Disk diffusion (**panel A**) and vapor phase (**panel B**) activity of TTO. MSSA: Methicillin-susceptibility *Staphylococcus aureus*; MRSA: Methicillin-resistant *Staphylococcus aureus*; ESBL: Extended Spectrum Beta-Lactamases; CS: Carbapenem-Susceptible; CR: Carbapenem-Resistant; Kp: *Klebsiella pneumoniae*; Ab: *Acinetobacter baumannii*; Pa: *Pseudomonas aeruginosa*.

The DD assay was used to evaluate the antimicrobial activity of the liquid phase in comparison to the analysis by VP assay used for the evaluation of the volatile compound activity. DD experiments showed a higher potency of TTO against Gram-negative bacteria than *S. aureus*. Regarding VP assays, the in-vitro effectiveness of TTO in VP was lower than that by DD analysis; nevertheless, TTO retained its activity against Gram-negatives, especially with regard to CR-Ab displaying 20 and 15 mm inhibition zone by means of DD and VP assays, respectively (Figure 1).

### 2.5. Time Kill Studies

**Figure 2:**
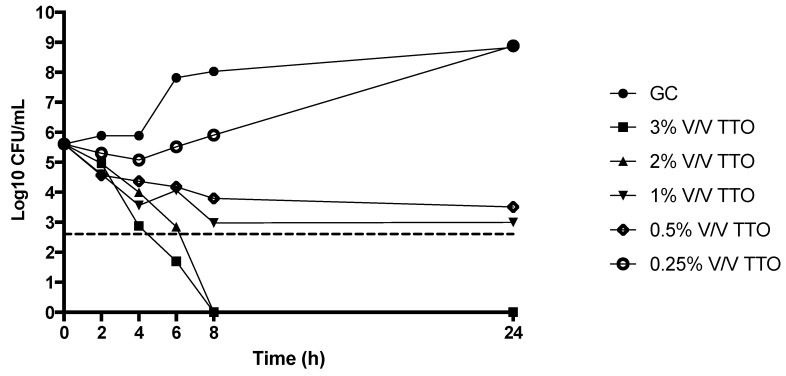
Activity of TTO against MRSA at different concentrations throughout killing study. GC: Growth Control; MRSA: Methicillin-resistant *Staphylococcus aureus*. Dashed line represents bactericidal activity.

In agreement with the MIC/MBC data, killing studies of TTO alone against MRSA showed a concentration-dependent effect, with an absence of bacterial growth at concentration of 2%; on the other hand, only a bacteriostatic effect was noted at the concentrations of 1% and 0.5% and no activity was observed at 0.25% (Figure 2).

**Figure 3:**
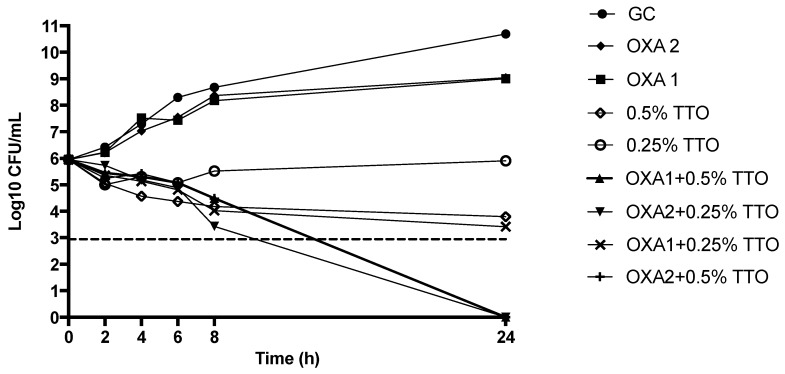
Activity of TTO (*v*/*v*) and OXA (μg/mL) at different concentrations against MRSA throughout killing study. GC: Growth Control; MRSA: Methicillin-resistant *Staphylococcus aureus*; OXA: oxacillin. Dashed line represents bactericidal activity.

Interestingly, TTO associated with OXA was shown to lower the antibiotic MIC against MRSA, which was resistant to all antimicrobial beta-lactams. Evaluation of bactericidal and synergistic activity of TTO combined with OXA was therefore conducted at sub-inhibitory concentrations (Figure 3). TTO and OXA alone at sub-inhibitory concentrations (0.25–0.5% and 1–2 μg/mL, respectively) were not able to reduce the bacterial amount, whereas the combinations of TTO + OXA at the same concentrations with the exception of 0.25% TTO + 1 μg/mL OXA, showed marked synergistic effects and bactericidal activity against MRSA, with absence of any bacterial growth at 24 h (Figure 3).

## 3. Discussion

The spread of multi-drug resistant bacteria is well recognized as an emergent global challenge, given the paucity of active therapeutical options and the high rate of mortality [2]. Thus, alternatives including natural substances are proposed as intriguing options for facing the problem of resistance in bacteria [24]. 

Based on these considerations, in the present study the antimicrobial activity of a commercial formulation of TTO was evaluated against *S. aureus* and several MDR Gram-negative bacteria. 

This report demonstrates a remarkable bactericidal activity of TTO against all the tested MDR Gram-negatives with absence of bacterial growth at concentrations ranging from 0.25% to 0.5% *v*/*v* for *E. coli*, ESBL-CS-Kp, CR-Ab, and CR-Kp whereas a lower bactericidal activity (1% *v*/*v*) was found for CR-Pa, in line with that observed in other studies and summarized by Carson et al., where most bacteria were susceptible to TTO at concentrations of 1.0% or less and higher MICs were reported for organisms such as *P. aeruginosa* [13].

To corroborate the TTO antimicrobial effectiveness, its synergistic activity was also investigated in combination with sub-inhibitory concentrations of traditional antimicrobials commonly used as a part of combination treatment against MDR Gram-negatives [25]. Among used antimicrobial MEM (to which all the CR strains were resistant) and COL (to which both CS and CR-Kp were resistant) a synergism was shown with all tested combinations, with FICI values lower than 0.5.

Other studies reported EOs’ synergistic activities when used in combination with antibiotics [26,27,28]; nevertheless, to the best of our knowledge, this paper demonstrates for the first time a potent antimicrobial activity of TTO, alone and in combination with different antimicrobials, against clinically relevant MDR Gram-negatives strains (i.e., CR-Kp, CR-Ab, and CR-Pa) and even PDR Kp, towards which the available therapeutic options are very limited or absent. In fact, previous studies investigated the activity of EOs other than TTO against ESBL producing Gram-negatives with unknown sensitivity to carbapenems [24] or oregano EO against CR-Kp with unknown sensitivity to colistin [29,30].

Thus, the results of the present study appear extremely promising for a potential clinical use of TTO in the setting of infections caused by MDR Gram-negative microorganisms. 

In addition, being aware that hospital pneumonia cases are usually caused by MDR Gram-negative bacteria [31], the potency of TTO was also evaluated in the VP [32]. Even though the VP efficacy was globally lower than that observed with the disk diffusion analysis (liquid phase), antibacterial activity was retained against *E. coli* and both CS and CR-Kp whereas no activity was found with regard to MSSA, MRSA, and CR-Pa. Interestingly, TTO in VP maintained an elevated activity towards CR-Ab, thus corroborating the results obtained by Miao Li et al in their model of pulmonary delivery of tea tree oil-b-cyclodextrin inclusion complexes [33]. It seems that no VP assays have yet been performed against other MDR and PDR Gram-negatives. If confirmed, the observed effectiveness of TTO VP against CR-Ab might have a critical clinical impact for the therapy of CR-Ab lung infection. 

Taking in to consideration these inspiring results, additional efforts should be undertaken for the implementation of TTO therapeutical use in the setting of MDR infections beyond cosmetics. 

However, the irritant properties and the hydrophobicity of TTO might limit its clinical application [34]. Although there have been reports of cutaneous and oral toxicity related to TTO, its general toxicology (i.e., LD_50_) profile suggested that severe reactions would be extremely rare in the absence of ingestion [35]. Others found the effect of TTO on cell viability as primarily dose dependent, with significant cytotoxicity at concentrations of ≥10% and ≥50% for cell lines and whole tissue, respectively [36].

To counterpart these limitations, herein it was observed that the remarkable antibacterial activity was present at very low concentration and, when combined with commonly therapeutically used antimicrobials, even at sub-inhibitory concentrations, thus making it reasonable to have further investigations on the best TTO percentage to be used for clinical purpose. On the other hand, a recent study performed in an animal model of bacterial and fungal pneumonia showed the efficacy of inhalable TTO nanoemulsion as a promising and intriguing local therapy to overcome difficulties in TTO formulation [37].

As well as for Gram-negative bacteria, local and systemic infections caused by MRSA represent a therapeutical challenge for physicians [38]. The present investigation showed that the addition of TTO at OXA sub-inhibitory concentrations was able to be reduced from 64 to 2 μg/mL its MIC, being OXA one of the more active beta-lactam against *S. aureus* but to which MRSA is typically resistant [39]. Killing studies confirmed that low concentrations of TTO (0.25–0.5% *v*/*v*) combined with OXA at concentrations equal to (2 μg/mL) and just lower than (1 μg/mL), the breakpoint for methicillin-susceptibility, obtained a potent concentration-dependent bactericidal and synergistic activity, with absence of bacterial growth after 24 h of antibiotic challenge. Although the MIC/MBC values of TTO against MSSA/MRSA were comparable with those obtained by a previous study [16], the killing results were slightly different, possibly due to the different concentrations used for the experiments (up to 3% compared with 5% *v*/*v*) [40]. Herein reported synergistic analyses confirmed the indifference between TTO and VAN, as previously described [15], whereas the novelty of this report is based on the observation (by both synergistic and killing studies) that the addition of TTO at sub-inhibitory concentration indeed restored MRSA sensitivity to OXA. Based on literature survey, no similar results have been yet described with TTO and MRSA and they appear to be crucial when considering the possibility of combining TTO with beta-lactams for MRSA infection. In fact, the activity of other EOs was evaluated against several MRSA strains [41,42] and the tendency of EOs to reduce antibiotic resistance was observed, with the combination natural compounds–synthetic drugs inducing the reversal of resistance in bacteria toward antibiotics such as penicillin [4], ampicillin/sulbactam [5], carbapenems [43], and oxacillin [44]. The mode of action of antimicrobial combination leading to the synergism is still an area of active research and it is probably attributed to the perturbation of bacterial membrane or the inhibition of PBP2a activity or its reduction [44] exerted by natural compounds. 

Given the anti MRSA activity, the potential clinical application of TTO might reside on the local administration of the drug (either at a fixed % *v*/*v* or vehiculated by the use of nanoparticles) for the treatment of skin and soft tissue infections, including those occurring after surgery [35,45] or nasal decolonization in the case of resistance to mupirocin or other treatments [46].

Because the reported differences in the antimicrobial activity of TTO could relate to the specific composition of the volatile compounds in the TTO, one of the strengths of this report was the combination of the chemical analysis of TTO components together with the evaluation of its antimicrobial activity both in liquid and VP. In particular, it has been reported that the antimicrobial activity of TTO is attributed mainly to terpinen-4-ol, which is the major component of the oil [47] and exhibits a favorable hydrophobic/hydrophilic profile [47]. Several studies on terpinen-4-ol have shown it to be a bactericidal agent. Ferrini et al. [48] reported its activity against strains resistant to mupirocin, fusidic acid, vancomycin, methicillin, and linezolid. In their article the terpinen-4-ol antistaphylococcal potency was found even higher than that of some antibiotics. In a different report the antimicrobial potency of some TTO chemical components was performed, including the terpinen-4-ol, by using disc diffusion and broth microdilution methods [13]. Terpinen-4-ol compared to the other components was found active against all tested microorganisms including *Escherichia coli*, *Candida albicans*, and *Staphylococcus aureus*. In the present study, the direct comparison of the two analyses (liquid and VP) indicated a marked increase in the percentage of α-pinene and a marked reduction in terpinen-4-ol percentage in the VP compared to that observed in the liquid phase. This difference could be responsible for the higher biological effects of liquid TTO, especially against Gram-positive bacteria. In addition, it is of note that the greatest effects were observed with eucalyptol, a component often considered to have marginal antimicrobial activity. Even in the present study, we confirmed that eucalyptol was highly represented in liquid as well as in VP, raising the possibility that even if eucalyptol may not be one of the primary antimicrobial components, it might contribute to the permeabilization of bacterial membranes and thus facilitate the entry of other and more active components [47]. 

As expected, head space analysis showed that the lightest components (α-pinene, β-pinene) were more represented than the heaviest ones (terpinen-4-ol, α-terpineol). This phenomenon was particularly evident for α-pinene, leading to the speculation that it might contribute to the high antibacterial activity of TTO in VP against CR-Ab. However, the presence of other trace components could also be important, given that they could act synergistically to exert the antimicrobial action.

Taken together, these findings support the hypothesis that TTO comprise a large number of components and it is likely that their mode of action involves several targets in the bacterial cell [49]. The observed different antimicrobial activity might depend on the different percentage of TTO components in liquid as well in VP assays, suggesting that additional investigations on the activity of a specific component against a specific microorganism should be further encouraged.

Finally, it should be noted that the TTO used in the present study was a commercial formulation; thus, the results herein described might be an expression of one specific TTO and not of all TTOs. Nevertheless, in order to overcome this limitation, the chemical composition of the used commercial formulation was widely investigated, both in liquid and VP. 

## 4. Materials and Method

### 4.1. Antimicrobials Agents and TTO

Antimicrobial agents were provided as purified powder by the manufacturer (Sigma Aldrich, Rome, Italy). Stock solutions at different concentrations were prepared in sterile and pyrogen-free 0.9% saline or water, according to the manufacturer’s instructions. The activity of the tested antimicrobials was expressed as μg/mL whereas the activity of TTO was expressed as %*v*/*v*. The used TTO was commercially acquired as a pure and natural commercial formulation (ESPERIS S. P. A. Milan, Italy).

### 4.2. TTO Chemical Composition Analysis

The GC/MS analysis was carried out with a GC-MS and GC-FID using a turbomass Clarus 500 GC-MS/GC-FID from Perkin Elmer instruments (Waltham, MA, USA). A Stabilwax fused-silica capillary column (Restek, Bellefonte, PA, USA) (60 m × 0.25 mm, 0.25 mm film thickness) was used with helium as carrier gas (1.0 mL/min). GC oven temperature was kept at 60 °C for 5 min and programmed to 220 °C at a rate of 5 °C/min and kept constant at 220 °C for 30 min. Solvent delay 0–2 min and scan time 0.2 s. MS was taken at 70 eV. Mass range was from 30 to 350 *m*/*z*. 1 μL of TTO was diluted in 1 mL of methanol and 1 μL of the solution was injected into the GC injector at a temperature of 280 °C.

To investigate TTO volatile component, a Perkin-Elmer Headspace (HS) Turbomatrix 40 autosampler connected to a Clarus 500 GC-MS was used for headspace analysis. To develop an optimal headspace procedure for the determination of volatile organic compounds (VOCs) from TTO, essential parameters such as equilibration time and temperature were adjusted.

The relative percentages for quantification of the components were calculated by electronic integration of the GC-FID peak areas. Identification of the constituents was performed based on MS library search (Nist MS Search Ver. 2.0 and Wiley 9th edition). Linear retention indices (LRI) of each compound were calculated using a mixture of aliphatic hydrocarbons (C8-C30, Ultrasci) injected directly into the GC injector with the same temperature program as reported above. Only chemical components with percentages higher that 0.1% were investigated (Table 1).

### 4.3. Bacterial Strains

For antimicrobial activity determination, we used the following microorganisms: MSSA (ATCC 29213), *E. coli* (ATCC 25922), MRSA (clinical strain isolated from skin), ESBL-CS-Kp (clinical strain isolated from urine), ESBL-CR-Kp (clinical strain isolated from urine), CR-Ab (clinical strain isolated from sputum) and CR-Pa (clinical strain isolated from bronchoalveolar lavage). 

After bacterial storage on cryovial bead preservation system (Microbank; Pro-Lab Diagnostics, Richmond Hill, ON, Canada) at −80 °C, inoculum was prepared by spreading one cryovial bead on blood agar plate and incubating overnight at 37 °C. One colony was re-suspended in 5 mL tryptic soy broth (TSB) and incubated at 37 °C without shaking. Overnight cultures were then adjusted to a turbidity of 0.5 McFarland, corresponding to ≈1 × 10^8^ CFU/mL.

### 4.4. Antimicrobial Activity

MIC and MBC of TTO, amikacin (AMK), cefazolin (CFZ), meropenem (MEM), oxacillin (OXA), colistin (COL), rifampin (RIF), and vancomicin (VAN) were determined by using the macro dilution broth method [50]. Briefly, two-fold serial dilutions of each antimicrobial agent and TTO were prepared in 2 mL Mueller Hinton broth (MHB) in borosilicate glass tubes and incubated for 24 h at 37 °C. MIC was defined as the lowest concentration of antibiotic that completely inhibited visible growth whereas bactericidal activity was defined as ≥3-log10 CFU/ml reduction of the initial bacterial count after 24 h of incubation. The used bacterial inoculum was ~5 × 10^5^ CFU/mL. 

### 4.5. Synergistic Activity of TTO Combined with Antimicrobial Agents

Checkerboard method was used to investigate the synergism of the following combinations: TTO + AMK/CFZ/OXA/VAN/RIF for MSSA/MRSA; TTO + AMK/MEM for *E. coli*; TTO + MEM/COL for CR-Ab; TTO + MEM/AMK/COL for CR-Pa; TTO + AMK/MEM/COL for ESBL-CS-Kp and CR-Kp. A 96-well microtitre plate containing TTO/antibiotic combinations at different concentrations (1–0.5–0.25% *v*/*v* and 0.5, 0.25, 0.125 × MIC, respectively) was used to perform checkerboard synergy testing. Wells containing a final inoculum of ~5 × 10^5^ CFU/mL were incubated at 37 °C for 24 h under static conditions in MHB. The fractional inhibitory concentration index (FICI) of each combination was defined as: ∑FIC: FICA + FICB = MICA + B/MICAalone + MICB + A/MICBalone. Synergism was defined as FICI ≤ 0.5 whereas FICI > 0.5 but <4 were considered as indifferent.

### 4.6. DD and VP Assay

The antimicrobial activity of TTO was compared against the same selection of antibiotic-resistant and -sensitive bacterial strains described above by using DD and VP assays.

For DD analysis, 10 μL of absolute (100% *v*/*v*) TTO were inoculated onto individual 6-mm filter paper discs and placed on Mueller Hinton Agar (MHA) plates containing ~1.5 × 10^8^ CFU/mL of the tested bacteria whereas for VP, TTO-impregnated (concentration as above) discs were placed on the lid of the petri dish and covered with parafilm, as previously described [51] so that only the TTO vapor fraction was responsible for organism inhibition.

The antimicrobial effect was assessed by measuring the inhibition zone (expressed as mm) after 24 h of incubation at 37 °C.

### 4.7. Time-Kill Studies

Given the unexpected effect of TTO in lowering OXA MIC against MRSA, which is resistant to all beta-lactam antimicrobials, the activity of TTO alone and in combination with OXA was evaluated by time-kill studies performed in the logarithmic growth phase using an initial inoculum of ~5 × 10^5^ CFU/mL.

Killing curves were performed in boro-silicate glass tubes in a final volume of 10 mL CAMHB which were further incubated at 37 °C. At 2, 4, 6, 8, and 24 h time points, 1 mL aliquots were sampled and washed with 0.9% saline solution in order to prevent the antibiotic carry-over effect. Ten-fold dilutions were then plated on Muller-Hinton agar and the number of CFUs was determined. Medium without antibiotics was used as growth control. Bactericidal activity was defined as ≥99.9% (i.e., ≥3-log10 CFU/mL) reduction of the initial bacterial count at each time point. Synergy was defined as a ≥100-fold decrease in CFU/mL between the combination and its most active constituent at the same concentration after 24 h, with the number of surviving organisms in the presence of the combination ≥100-fold CFU/mL below the starting inoculum. 

For TTO alone we used 3%, 2%, 1%, 0.5%, and 0.25% *v*/*v* whereas when tested in combination with OXA, we used the following concentrations: OXA 2 μg/mL (0.06 × MIC), OXA 1 μg/mL (0.03 × MIC), OXA 2 μg/mL (0.06 × MIC) + TTO 0.5%, OXA 2 μg/mL (0.06 × MIC) + TTO 0.25%, OXA 1 μg/mL (0.03 × MIC) + TTO 0.25%, OXA 1 μg/mL (0.03 × MIC) + TTO 0.25%.

All in-vitro experiments were performed in duplicate.

## 5. Conclusions

In conclusion (a) for the first time a significant potency of TTO (alone and in combination) was demonstrated against a selection of clinically relevant MDR/PDR Gram-negative microorganisms; (b) the antibacterial evaluation of TTO was performed both in liquid and VP, making it reasonable to be used as a possible inhalatory for lung infections, especially those caused by CR-Ab; (c) the activity of TTO/OXA combination against MRSA at sub-inhibitory concentrations might be expression of a restored susceptibility to OXA induced by TTO, with obvious clinical implications.

Taken together, the herein reported results might provide the basis for a possible role of TTO as part of non-conventional regimens against both MSSA/MRSA and Gram-negative MDR/PDR microorganisms. However, since several different varieties of TTO have been described so far [52] and given that in the present study only one type of TTO was thoroughly investigated, the results of the present research might be considered as a potential starting-point for additional studies on the activity of TTO against multi-drug resistant bacteria.

## Figures and Tables

**Table 1:** Chemical composition (%) of TTO.

# ^1^	Component ^2^	LRI ^3^	LRI_lit_^4^	A1% ^5^	A2% ^6^
**1**	α-pinene	1040	1039	12.4	22.5
**2**	β-pinene	1131	1124	1.8	2.4
**3**	1,4-cineole	1192	1192	0.5	-
**4**	α-terpinene	1197	1195	2.8	2.9
**5**	d-limonene	1216	1219	2.3	2.8
**6**	eucalyptol	1231	1230	15.2	16.5
**7**	γ-terpinene	1266	1265	9.8	10.7
**8**	o-cymene	1291	1287	6.3	8.5
**9**	terpinolene	1306	1299	1.6	1.6
**10**	aromadendrene	1600	1603	1.9	-
**11**	terpinen-4-ol	1631	1633	35.4	28.7
**12**	α-terpineol	1718	1724	8.1	3.4
**13**	ledene	1715	1707	1.1	-
**14**	globulol	2110	2104	0.8	-
**Total**				100	100

^1^: compound identification number; ^2^: components are listed according to their elution order on a polar column. ^3^: Linear Retention indices measured on a polar column; ^4^: Linear Retention indices from literature; ^5^: Area by standard GC-MS (%); ^6^: Area by Head Space GC-MS (%). Only chemical components with percentages greater than 0.1% were included.

**Table 2:** Antibacterial activity of TTO and different antimicrobials against MSSA, MRSA, *Escherichia coli*, ESBL-CS-Kp, ESBL-CR-Kp, CR-Ab, and CR-Pa.

Strains	TTO ^1^		AMK ^2^		OXA ^3^		CFZ ^4^		VAN ^5^		RIF ^6^		MEM ^7^		COL ^8^	
	MIC ^9^	MBC ^10^	MIC	MBC	MIC	MBC	MIC	MBC	MIC	MBC	MIC	MBC	MIC	MBC	MIC	MBC
	% *v*/*v*		μg/mL		μg/mL		μg/mL		μg/mL		μg/mL		μg/mL		μg/mL	
MSSA ^11^	1	2	4	8	0.25	0.50	0.50	0.50	0.50	1	0.007	0.007	NA		NA	
MRSA ^12^	0.50	2	32	32	32	64	64	128	1	1	0.007	0.007	NA		NA	
*E. coli* ^13^	0.25	0.25	4	4	NA ^20^		NA		NA		NA		0.060	0.060	0.50	0.50
ESBL-CS-Kp ^14,15,16^	0.50	0.50	0.50	0.50	NA		NA		NA		NA		0.125	0.250	256	256
ESBL-CR ^17^	0.25	0.25	64	64	NA		NA		NA		NA		256	512	128	128
CR-Ab ^18^	0.25	0.25	8	16	NA		NA		NA		NA		64	128	0.25	0.25
CR-Pa ^19^	1	1	8	8	NA		NA		NA		NA		8	16	1	2

^1^: Tea Tree Oil; ^2^: amikacin; ^3^: oxacillin; ^4^: cefazolin; ^5^: vancomycin; ^6:^ rifampin; ^7^: meropenem; ^8^: colistin. ^9^: Minimal Inhibitory Concentration; ^10^: Minimal Bactericidal Concentration; ^11^: Methicillin-susceptibility *Staphylococcus aureus*; ^12^: Methicillin-resistant *Staphylococcus aureus*; ^13^: *Escherichia coli*; ^14^: Extended Spectrum Beta-Lactamases; ^15^: Carbapenem-Susceptible; ^16^: Kp; ^17^: Carbapenem-Resistant; ^18^: *Acinetobacter baumannii*; ^19^: *Pseudomonas aeruginosa*; ^20^: Not Active.

**Table 3:** Qualitative assessment of synergistic activity by FICI * values of TTO combined with different antimicrobials against MSSA, MRSA; *Escherichia coli*, ESBL-CS-Kp, ESBL-CR-Kp, CR-Ab, and CR-Pa.

Strains	TTO ^1^ + AMK ^2^	TTO + OXA ^3^	TTO + CFZ ^4^	TTO + VAN ^5^	TTO + RIF ^6^	TTO + MEM ^7^	TTO + COL ^8^
MSSA ^9^	0.25	0.32	0.25	>0.5	0.32	-	-
MRSA ^10^	0.20	0.32	0.32	>0.5	0.32	-	-
*E. coli* ^11^	0.25	NA ^18^	NA	NA	NA	>0.50	0.13
ESBL-CS-Kp ^12,13,14^	>0.50	NA	NA	NA	NA	0.50	0.32
ESBL-CR-Kp ^15^	0.50	NA	NA	NA	NA	0.32	0.32
CR-Ab ^16^	0.32	NA	NA	NA	NA	0.32	0.21
CR-Pa ^17^	0.25	NA	NA	NA	NA	0.50	0.25

*: FIC index; ^1^: Tea Tree Oil; ^2^: amikacin; ^3^: oxacillin; ^4^: cefazolin; ^5^: vancomycin; ^6:^ rifampin; ^7^: meropenem; ^8^: colistin.; ^9^: Methicillin-susceptibility *Staphylococcus aureus*; ^10^: Methicillin-resistant *Staphylococcus aureus*; ^11^: *Escherichia coli*; ^12^: Extended Spectrum Beta-Lactamases; ^13^: Carbapenem-Susceptible; ^14^: Kp; ^15^: Carbapenem-Resistant; ^16^: *Acinetobacter baumannii*; ^17^: *Pseudomonas aeruginosa*; ^18^: Not Applicable.

## Data Availability

The data used to support the findings of this study are available from the corresponding author upon request.

## Abbreviations

MDR	multidrug-resistant
CR	carbapenem-resistant
EOs	essential oils
TTO	Tea tree oil
MSSA	methicillin-susceptible *Staphylococus aureus*
MRSA	methicillin-resistant *Staphylococus aureus*
ESBL	extended-spectrum beta lactamases
CS	carbapenem-sensitive
Kp	*Klebsiella pneumoniae*
Ab	*Acinetobacter baumannii*
Pa	*Pseudomonas aeruginosa*
HS	Headspace
VOCs	volatile organic compounds
LRI	Linear retention indices
TSB	tryptic soy broth
MIC	minimal inhibitory concentration
MBC	minimal bactericidal concentration
AMK	amikacin
CFZ	cefazolin
MEM	meropenem
OXA	oxacillin
COL	colistin
RIF	rifampin
VAN	vancomicin
MHB	Mueller Hinton Broth
FICI	fractional inhibitory concentration index
DD	disk diffusion
VP	vapour phase
MHA	Mueller Hinton Agar
CFUs	Colony Forming Units
PDR	pan-drug resistant
